# Factors causing delayed presentations of breast cancer among female patients in Sulaimani Governorate, Kurdistan region, Iraq

**DOI:** 10.1186/s12905-023-02656-x

**Published:** 2023-11-16

**Authors:** Alaa Abdulrazzaq Abdulkareem, Hawar Ali Ghalib, Mezjda Ismail Rashaan

**Affiliations:** 1 Department of Surgery, College of Medicine, University of Sulaimani, Sulaimaniyah, Iraq; 2 Department of Breast Diseases Treatment, Shar Teaching Hospital, Sulaimaniyah, Iraq

**Keywords:** Breast cancer, Early detection, Late presentation, Cross-sectional study, Health behaviours

## Abstract

**Background:**

Since breast cancer (BC) has the best chance of being effectively treated when it is tiny and has not spread, encouraging early disease detection remains a fundamental goal in improving prognosis.

**Objective:**

To quantify the magnitude of the delay in BC presentation as well as the contributing factors related to this delay.

**Patients and methods:**

Data was collected at the Breast Diseases Treatment Clinic, Shar Teaching Hospital, Sulaimani, Iraq from January 2017 to December 2021 of 429 patients. A validated questionnaire was distributed to women about their demographics, health, and general awareness of the disease. The patient delay was calculated by collecting information on when they initially began experiencing symptoms and when they saw a doctor. We also gleaned information about the tumour’s clinicopathological features from the patient’s medical records.

**Results:**

The participants’ ages ranged from 24 to 85 years, with a mean of 49.6 ± 11. Most women were middle-aged (53.8%), from urban areas (80.2%), illiterate (41.7%), married (86.5%), housewives (79.0%), given birth to > 4 children (34%), practised breastfeeding (78.8%), and non-smokers (91.4%). Regarding patients’ health behaviours, there were highly significant correlations between early and late presentation (p < 0.001). Concerning the patient’s awareness of BC symptoms, there were highly significant correlations between early and late presentation (p < 0.001). In addition, the late presentation was strongly correlated with bilateral BC, lymph node involvement, inflammatory BC, grade III BC, and T4 status.

**Conclusions:**

Our findings shed light on possible causes of late presentation and identified those at risk of delayed consultation. Our communities need to be educated about BC, and encouraging them for early detection decreases the incidence of advanced BC.

## Introduction

The second leading cause of death for women globally, after lung cancer, is BC, which affects more women than any other type of cancer. In 2020, it was assessed that 2.3 million females were diagnosed with breast cancer representing 11.7% of all cancer cases, and 685,000 deaths representing 6.9% of all cancer deaths in females [1]. From 2000 to 2019, the number of BC new cases in Iraq increased from 24.36 to 52.7 per 100,000 people [2]. About 69.5% of the patients were presented with stage 2 or 3 of the disease, in the first, or localized stage, 4.1 percent of individuals were diagnosed, and 18.2% of patients were referred to oncological services with insufficient information for a clinical-stage to be assigned [3].

BC fatalities have been rising in low-income countries while falling in high-income countries in recent years. This disparity can be attributed to cancer being diagnosed later in low-income countries [4–6]. A delay in obtaining help for BC symptoms may contribute to a more advanced stage and a larger tumour. Patients’ delay refers to the time that passes between when a patient experiences a symptom for the first time and when she sees a doctor [7, 8]. According to a British study, patient delay among women with BC symptoms was a contributing factor to the disease progression [9].

It is well known that patients who delay the onset of their disease in the clinic by 12 to 26 weeks have lower survival chances than those who delay it by < 12 weeks [10]. Time lags vary considerably from one country to another. When compared to studies conducted in the United Kingdom (13 days) and New Zealand (14 days), the patient delay in Iraq was 30 days [11].

Studies have shown that patients’ socioeconomic status, level of education, beliefs, and conditional health behaviour play a role in their procrastination when seeking medical care [8, 12]. Awareness of BC has a major impact on the prevalence and survival rates of the disease [13]. BC education provided to the Estonian public one year before the onset of symptoms reportedly increased the proportion of cases diagnosed early or reduced patient delay [8].

BC screening, early diagnosis and down-staging programs were first offered at the Breast Diseases Treatment Clinic, Shar Teaching Hospital, Sulaimaniyah, Iraq, in 2007 [14]. Since more studies are needed to examine the causes of delay. So, this study aimed to compare awareness of BC warning signs between different groups of patients and to examine the effect of symptom patterns on the length of presentation; as well as to ascertain the impact of late presentation on the tumor’s clinicopathological characteristics.

## Patients and methods

### Populace and scrutiny sites

This retrospective study was conducted from January 2017 to December 2021 at the Breast Disease Treatment Clinic, Shar Teaching Hospital, Sulaimani, Iraq on 429 women who had been diagnosed with BC based on a breast physical examination, mammography, and histology (for more confirmation).

### Inclusion criteria

Patients diagnosed with primary BC were included regardless of age, nationality, and treatment status.

### Exclusion criteria

Patients who had breast metastases from another primary cancer were excluded from the study. Additionally, patients with incomplete medical records and cognitive issues that affected their memory or communication skills to the extent that they could not give consent were also not included.

### Questionnaire and data collection

A structured and validated questionnaire that was adapted from reviews of various literature that could answer the purpose of the study was used to collect the data, either by face-to-face interviews or phone interviews. In some instances, data were gathered from first-degree relatives. The average time required to collect data from patients was approximately 10 min. The questionnaire addressed various factors associated with the delayed presentation of BC, including sociodemographic information (age, residency, marital status, occupation, educational level, number of children, breastfeeding status, age at menarche, height, and weight). Data also include health conditions, a family history of BC, the first clinical symptoms, breast self-examination, a previous visit to a doctor for a breast problem, and breast surgery. In addition, the medical records were looked at to determine the size of the tumour when it was first found, its laterality, its spread to lymph nodes, and its histological type and grade.

### Study protocol

In a single segment, individuals’ awareness of BC symptoms was evaluated. The BC symptoms were modified from the basic Breast Cancer Awareness Measure (BCAM) [15]. G* program was used to calculate sample size and participants responded yes, no, or didn’t know the questions. The length of symptoms was used to figure out “patient delay”. The most common threshold for figuring out “patient delay” was 3 months. Patients were split into two groups; those with “early presentation,“ which meant that they were screened or saw a doctor within 3 months of when their symptoms started, and those with “late presentation,“ which told that it had been > 3 months. We used a delay of > 3 months to characterize patient delay because there is substantial evidence that such delays are associated with poor survival.

### Statistical analysis

All submitted questionnaires were assigned unique codes and numbers for easy tracking. Microsoft Excel was used to insert the data, and then the spreadsheet was exported to the Statistical Package for Social Science (SPSS, IBM, USA, Version 26) for further analysis. Additionally, Pearson Chi-Square was utilized to ascertain the significance of the connection between the various combinations of independent and dependent variables. P-value set as highly significant (p ≤ 0.001), significant (p ≤ 0.05), non-significant (p ≥ 0.05).

## Results

### Scrutiny populace

The patients’ mean ± SD age was 49.6 ± 11.8 years with an age range of 24 to 85 years. Most patients (53.8%) were aged 41–55 years, lived in an urban area (80.2%), were illiterate (41.7%), married (86.5%), housewives (79.0%), had > 4 children (34%), breastfed (78.8%), and non-smokers (91.4%). The mean ± SD age at menarche was 13.17 ± 1.5 years (404/429 patients answered this question), and 414/429 participants had their weight and height recorded at the time of diagnosis with a body mass index (BMI) mean of 29.89 ± 4.8 kg/m^2^ (Table 1).

**Table 1:** Sociodemographic characteristics of studied participants

Variable		Frequency	Percentage
Age (Year)	24–40	88	20.5
	41–55	231	53.8
	56–70	81	18.9
	> 70	29	6.8
Residency	Urban	344	80.2
	Rural	86	19.8
Educational status	Illiterate	179	41.7
	Primary School	113	26.3
	Secondary School	71	16.6
	Institute	20	4.7
	University or higher	46	10.7
Marital status	Single	39	9.1
	Married	371	86.5
	Widow or divorce	19	4.4
Occupation	Housewife	339	79.0
	Employed	78	18.2
	Unemployed	2	0.5
	Retired	9	2.1
	Student	1	0.2
Parity	Nulliparous	62	14.5
	One	26	6.1
	Two	63	14.7
	Three	72	16.8
	Four	60	14.0
	>Four	146	34.0
Breastfeeding	Yes	338	78.8
	No	91	21.2
Smoking status	Yes	21	4.9
	Ex–smoker	16	3.7
	No	392	91.4
Total		429	100.0

Moreover, the majority of women (77.4%) present with no history of benign breast disease, 47 (11.0%) had a history of retracted nipples, 26 (6.1%) had a history of psychological illness, 63 (14.7%) had a family history of BC, 62 (14.5%) had a family history of other malignancies, 28 (6.5%) had a history of breast surgery, 31 (7.2%) had a history of breast biopsies.

Regarding the patient’s health behaviours associated with delayed breast cancer presentation, only 108 (25.2%) patients underwent a mammogram in the two years preceding their diagnosis, 161 (37.5%) conducted breast self-examination, 253 (59%) showed a clinical breast examination, 111 (25.9%) expressed worry about having cancer and 95 (22.1%) indicated being affected by others. Consequently, there were highly significant correlations between screening and early presentation (≤ 3 months) and late presentation (> 3 months) (p < 0.001) (Table 2).

**Table 2:** Patient’s health behaviors associated with delay breast cancer presentation

Health behavior		Duration of symptoms		Total	P-value
		Screening and early presentation (≤ 3 months)	Late presentation (> 3 months)		
		Number, %			
Breast self-examination	Yes	73 (30.4)	88 (46.6)	161 (37.5)	< 0.001**
	No	167 (69.6)	101 (53.4)	268 (62.5)	
Clinical breast examination	Yes	203 (84.6)	50 (26.5)	253 (59.0)	< 0.001**
	No	37 (15.4)	139 (73.5)	176 (41.0)	
Mammography	Yes	98 (40.8)	10 (5.3)	108 (25.2)	< 0.001**
	No	142 (59.2)	179 (94.7)	321 (74.8)	
Fearful of having cancer	Yes	38 (15.8)	73 (38.6)	111 (25.9)	< 0.001**
	No	202 (84.2)	116 (61.4)	318 (74.1)	
Influenced by other	Yes	15 (6.3)	80 (42.3)	95 (22.1)	< 0.001**
	No	225 (93.7)	109 (57.7)	334 (77.9)	
Total		240 (100.0)	189 (100)	429 (100)	

**: Highly significant difference

Concerning the patient’s awareness of BC symptoms, only 331 participants completed the questionnaire, and 98 had missing data. Breast lump was the most commonly recognized symptom of BC in 295 patients (89.1%), followed by nipple discharge in 241 patients (72.8%) and an armpit lump in 228 (68.2%), while the least recognized symptoms were a change in the breast size in 133 patients (40.2%), nipple rash with change in the shape of the breast in 170 women (51.4%). Consequently, there were highly significant correlations between screening and early presentation (≤ 3 months) and late presentation (> 3 months) (p < 0.001) (Table 3).

**Table 3:** Patient’s awareness of breast cancer symptoms

Awareness		Duration of Symptoms		Total	P-value
		Screening and early presentation (≤ 3 months)	Late Presentation (> 3 months)		
Nipple shape change	Yes	162 (73.8)	52 (43.3)	214 (64.7)	< 0.001**
	No	36 (17.1)	56 ( 46.7)	92 (27.8)	
	I don’t know	13 (6.2)	12 ( 10.0)	25 (7.6)	
Nipple discharge	Yes	178 (84.4)	63 (52.5)	241 (72.8)	< 0.001**
	No	20 (9.5)	36 (30)	56 (16.9)	
	I don’t know	13 (6.2)	21 (17.5)	34 (10.3)	
Breast lump	Yes	205 (97.2)	90 (75.0)	295 (89.1)	< 0.001**
	No	1 (0.5)	6 (5.0)	7 (2.1)	
	I don’t know	5 (2.1)	24 (20.0)	29 (8.8)	
Breast size change	Yes	103 (48.8)	30 (25.0)	133 (40.2)	< 0.001**
	No	85 (40.3)	73 (60.8)	158 (47.7)	
	I don’t know	23 (10.9)	17 (14.2)	40 (12.1)	
Breast shape change	Yes	135 (64.0)	35 (29.2)	170 (51.4)	< 0.001**
	No	54 (25.6)	71 (59.2)	125 (37.8)	
	I don’t know	22 (10.4)	14 (11.7)	36 (10.9)	
Nipple rash	Yes	89 (42.2)	23 (19.2)	170 (51.4)	0.003*
	No	94 (44.5)	83 (69.2)	125 (37.8)	
	I don’t know	28 (13.3)	14 (11.7)	36 (10.9)	
Lump under armpit	Yes	175 (82.9)	53 (44.2)	228 (68.9)	< 0.001**
	No	15 (7.1)	36 (30.0)	51 (15.4)	
	I don’t know	21 (10.0)	31 (25.8)	52 (15.7)	
Total		211 (100)	120 (100)	331 (100)	

*: Significant difference, **: Highly significant difference

**Fig. 1:**
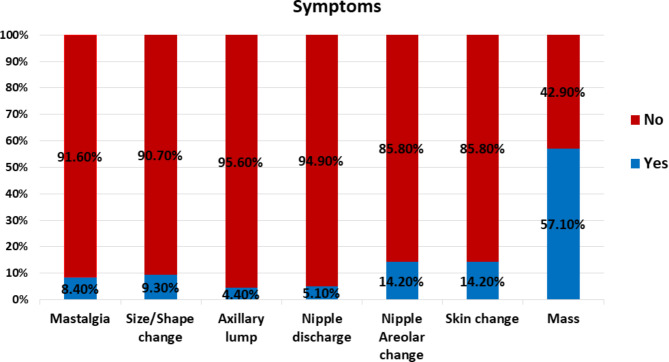
First presenting symptom of the studied participants

Moreover, a lump was the primary presenting complaint in most patients (57.1%), painless in 47.6% and painful in 9.5%. Other symptoms, including skin/nipple areola change (14.2%), breast size/shape change (9.3%), nipple discharge (5.1%), axillary lump (4.4%), and mastalgia (8.4%) were also found (Fig. 1).

**Fig. 2:**
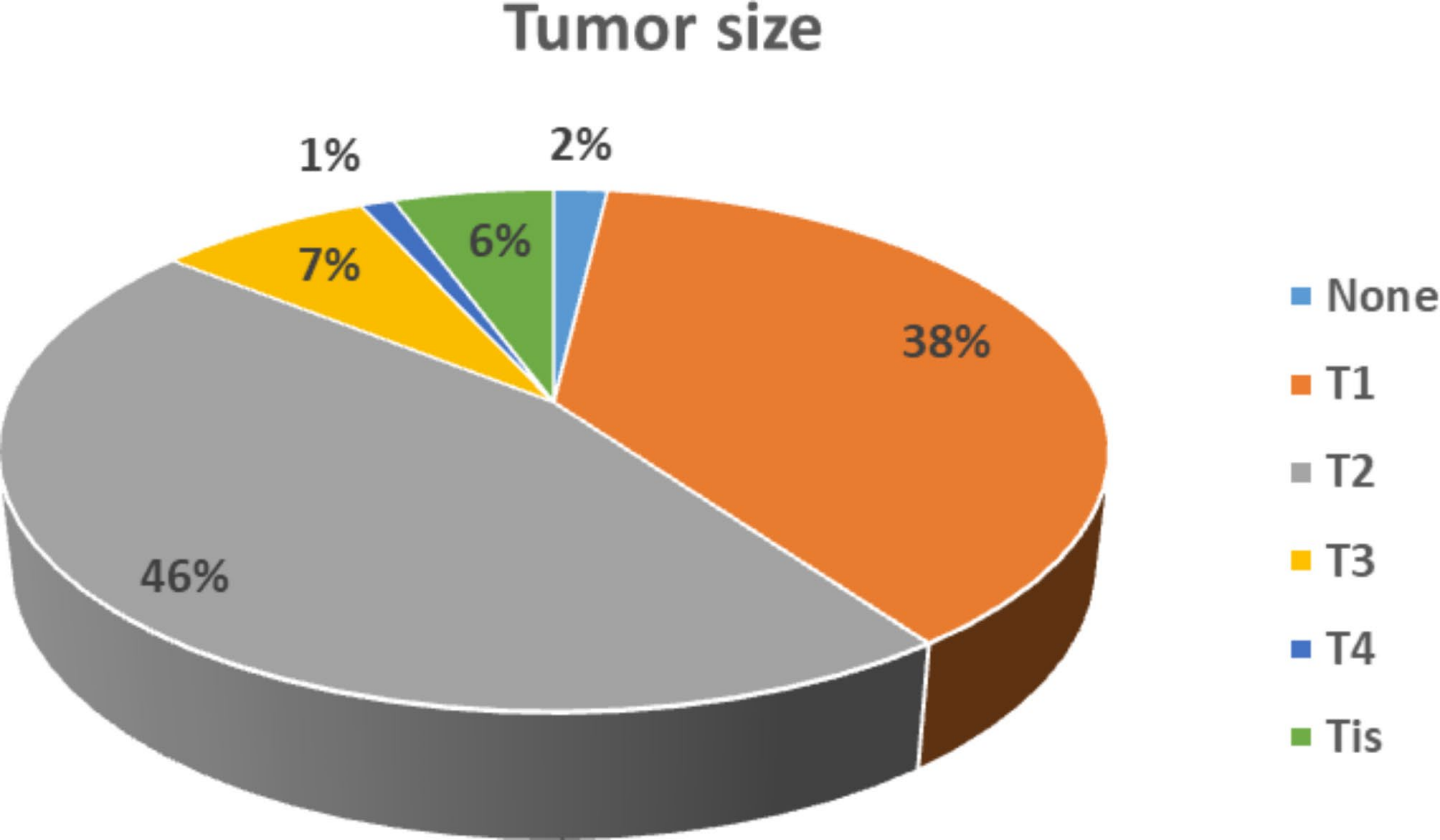
Tumor size of the studied participants

On the other hand, we categorized patients according to how long they had been experiencing symptoms and during screening and we found that 62 (14.5%) patients experienced < 1 month; 135 (31.5%) experienced 1–3 months; 122 (28.4%) experienced 4–6 months, 67 (15.6%) experienced > 6 months, and 43 (10.0%) during screening. Simultaneously, most patients (no.=201, 46.9%) were diagnosed with left-sided BC, lymph node involvement (no.=231, 53.8%), invasive ductal carcinoma (IDC) (no.=353, 82.5%), grade II (no.=224, 52.3%), and in situ was presented in 284 (66.2%) participants (Table 4). Regarding the tumour size in studied patients, it was T2 in 196 patients (45.7%), followed by T1 in 164 women (38.2%), then T3 in 32 patients (7.5%) and Tis in 24 patients (5.6%) with T4 only in 5 participants (1.2%) (Fig. 2).

**Table 4:** Effect of presentation delay on clinicopathological features of breast cancer at diagnosis

Tumor clinicopathological features		Duration of symptoms (Month)					Total	P-value
		Screening	< 1	1–3	4–6	> 6		
		Number, %						
Laterality	Left breast	19 (44.2)	26 (41.9)	61 (45.2)	66 (54.1)	29(43.3)	201(46.9)	0.02*
	Right breast	24 (55.8)	35(56.5)	74(54.8)	54(44.3)	33(49.3)	220(51.3)	
	Bilateral	0 (0)	1 (1.6)	0 (0)	2 (1.6)	5 (7.5)	8(1.9)	
Lymph node involvement	Yes	1 (2.3)	6 (9.7)	11 (8.2)	22(18.0)	16(23.9)	56 (13.1)	0.002*
	No	42 (97.7)	56(90.3)	123(91.8)	100(82.0)	51(76.1)	372(86.9)	
Histological type	IDC	32 (74.4)	51 (82.3)	108 (80.6)	10 (85.2)	58(86.6)	353(82.5)	0.02*
	ILC	3 (7.0)	2 (3.2)	12 (9.0)	8 (6.6)	6 (9.0)	31 (7.2)	
	IDC with ILC	0 (0)	0 (0)	0 (0)	2 (1.6)	0(0)	2 (0.5)	
	In Situ	7 (16.3)	8 (12.9)	9 (6.7)	1 (0.8)	0 (0)	25 (5.8)	
	Inflammatory Breast Cancer	0 (0)	0 (0)	0 (0)	1 (0.8)	2 (3.0)	3 (0.7)	
	Pagets’ disease	0 (0)	0 (0)	0 (0)	2 (1.6)	1 (1.5)	3 (0.7)	
	Mucinous Cancer	1 (2.3)	0 (0)	1 (0.7)	3 (2.5)	0 (0)	5 (1.2)	
	Papillary Cancer	0 (0)	1 (1.6)	3 (2.2)	1 (0.8)	0 (0)	5 (1.2)	
	Phyloid Tumor	0 (0)	0 (0)	1 (0.7)	0 (0)	0 (0)	1 (0.2)	
In situ	Present	41 (95.3)	49 (79)	10 (74.6)	62 (50.8)	32(47.8)	284(66.4)	< 0.001**
	Not seen	2 (4.7)	13 (21)	34 (25.4)	60(49.2)	35(52.2)	144(33.6)	
Histological grade	G I	2 (4.7)	4 (6.5)	14 (10.4)	6 (4.9)	2 (3.0)	28 (6.5)	< 0.001**
	G II	30 (69.8)	40(64.5)	74 (55.2)	53 (43.4)	27(40.3)	224(52.3)	
	G III	11 (25.5)	18(29.0)	46 (34.3)	63 (51.6)	38(56.7)	176(41.1)	
Tumor size	None	0 (0)	3 (4.8)	2 (1.5)	2 (1.6)	1 (1.5)	8 (1.9)	< 0.001**
	T 1	35 (81.4)	31(50.0)	75 (55.6)	20 (16.4)	3 (4.5)	164 (38.2)	
	T2	2 (4.7)	19(30.6)	49 (36.3)	84 (68.9)	42(62.7)	196(45.7)	
	T3	0 (0)	0 (0)	2 (1.5)	14 (11.5)	16(23.9)	32 (7.5)	
	T4	0 (0)	0 (0)	0(0)	1 (0.8)	4 (6.0)	5 (1.2)	
	Tis	6 (14.0)	9 (14.5)	7 (5.2)	1 (0.8)	1 (1.5)	24 (5.6)	
Total		43 (100)	62 (100)	135(100)	122(100)	67 (100)	429(100)	

*: Significant difference, **: Highly significant difference, IDC: Invasive ductal carcinoma, ILC: Invasive lobular carcinoma

### Considerations attributable to presentation delays

Screening and a 3-month or less interval between symptom discovery and first presentation for medical care are considered “early presentation”, while > 3 months was considered “late” or “delayed” presentation. About 240 (56%) participants exhibited early, whereas the remaining 189 (44.0%) presented late. There was a clear correlation between age and late presentation; younger women waited longer than older women before presenting their symptoms to a physician. Of 88 women aged < 41, 51 were present late (p < 0.001). Rural women (p < 0.001) and women with a poor level of education (p < 0.001) were at high risk for late presentation. Housewives were significantly more likely than other women to present late (no.=164, 86.8%) (p < 0.001). About 229 (95.4%) women who showed early had never smoked (p = 0.003). Other sociodemographic characteristics were not significantly related to patient delay (Table 1). Obesity was strongly associated with the late presentation; 73/189 (40.3%) women with the late presentation were obese, 58 (32.0%) were overweight, 41 (22.7%) were normal, and 9 (5.0%) had morbid obesity (p = 0.002). Significantly (p < 0.001), more patients with a late presentation had a history of benign breast disease (89.9%) and without a family history of BC (94.7%). In contrast, women with a first- or second-degree relative with BC were likelier to have an early presentation (p < 0.001). Women with no history of breast surgery or biopsy had an elevated risk of late presentation (p = 0.01).

Women who had not performed breast self-examination before their condition and those who performed clinical breast examinations regularly were more likely to present within 3 months (p < 0.001). In contrast, the risk of late presentation was high among those with no history of mammograms, feared having cancer, and were influenced by others (p < 0.001) (Table 2).

### Awareness of BC symptoms and late presentation

Late-presenting women were likelier to be unaware of BC symptoms than early-presenting women (120 vs. 211). According to Table 3, there was a greater risk of late presentation among women who could not distinguish nipple shape change, breast size change or breast shape change (p < 0.001). Inadequate knowledge of nipple rash as a BC symptom was also a significant factor associated with a higher probability of late presentation (p = 0.003). Patients who were aware of nipple discharge, breast lump, and armpit lump as BC symptoms were much more likely to present early presentations (p < 0.001).

### First presenting symptom and duration of symptoms

When patients were asked why they did not visit a medical facility earlier, the most common response was that they did not believe the initial symptoms to be severe. Among women who presented late, 67.7% experienced a painless mass as their first symptom (p = 0.001). Women with a change in the size or shape of the breast also had an increased probability of presenting late (p = 0.04). In contrast, the prevalence of nipple-areolar changes as a first symptom was considerably higher in women who reported a delay of > 3 months (p = 0.007). Women with skin changes like dimpling as their first symptom had a marginally increased chance of late presentation (p = 0.04). Mastalgia was substantially more prevalent in women who presented early (p = 0.001). Similarly, nipple discharge was considerably more common among women who presented early (p = 0.02). There was no significant difference (p = 0.11) between the armpit lump as the initial symptom.

### Effect of delay on BC clinicopathological features

More than 3 months of presentation delay was associated with an increased risk of bilateral BC (p = 0.02) and lymph node involvement (p = 0.001). Inflammatory BC and Paget’s disease were only observed in patients with a delay of 4–6 months and > 6 months (p = 0.02). There was a significant relationship (p < 0.001) between early presentation and the presence of in situ at diagnosis. Patients with a delay in presentation of > 6 months were more likely to present with grade III (p < 0.001). There was a significant positive correlation between tumour size and symptom duration. T4 was only present in women who presented 4–6 months or > 6 months late, but the majority of Tis was detected in both women who came for screening and those who came earlier than a month (p < 0.001) (Table 4).

## Discussion

This study assessed the delayed presentation of 429 women with BC and its associated factors at a Breast Disease Treatment Center in Sulaimani Governorate, Kurdistan Region, Iraq. The complex interplay of numerous causes leads to delayed presentation, a critical health hazard for women with BC. With each delay, the likelihood of presenting with advanced disease and the associated increased mortality risk increased dramatically. Despite the constant discovery of novel and more effective therapeutic modalities for BC, the disease’s frequency appears to be increasing worldwide, and it remains the top cause of mortality [16]. One of the most reliable causes of late diagnosis and poor prognosis is a delay in the onset of symptoms. Compared to women in the West, women in Iraq are diagnosed with BC at a far earlier age (roughly ten years), and 69.5% were already at an advanced clinical stage when detected [17].

In line with earlier research conducted in Kurdistan, Iraq, our study found that women had a mean age of 49.6 ± 11.8 years [18, 19]. The mean age was 47.4 ± 11 years and 49.4 ± 12.1 years, as reported by Majid et al. and Karim et al., respectively [3, 17]. The participants’ ages in the other study [10] were between 25 and 64 years, with a mean age of 46.4 ± 11.1 years.

Our study found that nearly half of all BC patients (44.0%) wait > three months before seeing a doctor for the first time, a percentage that is relatively higher than that of the United States (17%) [20], and Europe (17.3%) [21]. It’s lower than other low- and middle-income nations like Pakistan (84%) [22], but on par with the Malaysian report (42.5%) [23]. The reasons why patient wait times vary from country to country might be related to the role of patients’ sociodemographics on the duration of their symptoms. In this study, there were several major indicators of late presentation, including young age, living in a rural area, having a poor level of education, obesity, being a housewife, and smoking.

Following a study from the United States [24], younger persons appear to delay longer, but a study from Germany indicates that older women are at greater risk for late presentation [7]. Several hypotheses have been advanced to explain why a patient delay is more prevalent among younger patients. First, young women may misunderstand early BC complaints as symptoms of benign breast diseases and may be unaware of their BC risk at this age.

Living in a rural area far from the specialized centre significantly predicted late presentation. Similar results were discovered in a study of Moroccan women [25]. Additionally, having a lower level of education (52.4% of our patients) or being a housewife (86.8%) demonstrated a potential predictor of late presentation. Additional research has proven the importance of education in reducing delays [26, 27]. According to the international study by Jassem et al., the delay was shorter for middle-aged women, employed women, and women residing in large urban areas [28].

Obese women are more likely to appear late due to the relationship between BMI and patient delay, as determined by our analysis. One possible reason is that they observe specific symptoms which are less noticeable in women with large breasts.

Most earlier studies of patient delays among people with BC didn’t look at how smoking affected patient delays, but a study in Estonia found that female smokers had longer delays [8]. In our study, smokers and ex-smokers were more likely than nonsmokers to present with a late presentation. The act of smoking may be a reflection of women’s beliefs regarding health-promoting behaviours. Consistently with earlier studies, we found no association between patient delay and age at menarche, marital status, parity, or breastfeeding [10, 11, 29].

Previous benign breast conditions were strongly related to a late presentation in our analysis. For those women in the past, physicians may have deemed identical breast tissue modifications noncancerous, which may be one of the reasons why these women delayed seeking medical attention. Therefore, it may be beneficial to advise women with known benign breast disease to promptly report new breast symptoms to a physician to avoid postponing a BC diagnosis. In studies conducted in Germany and Estonia, the history of benign mastopathy was also revealed to be a factor in extended patient delays [7, 8].

Our survey subjects who did not have a family history of BC had an increased chance of presenting later. In contrast, women with first- or second-degree relatives were more likely to present earlier. Women with a family history of BC may be more breast-aware and less apprehensive of treatment, resulting in earlier care seeking.

We are unaware of any previous research that has explored the association between a history of breast surgery or biopsy and late presentation. We noticed that 22 out of 28 women with a history of breast surgery and 26 out of 31 women with a history of breast biopsy obtained medical support promptly following the onset of their first symptom. One of the reasons why these ladies may be so prompt to seek medical attention is that women who have faced a similar situation may be more conscientious and cautious about their health than women who have never encountered a similar problem. There was no link between delayed presentation and a history of retracted nipples, psychological factors, or other cancers in the family.

Contrary to predictions and other studies [30, 31], we found that an absence of breast self-examination was associated with an increased chance of early presentation. One of the reasons why women performing breast self-examination may appear late is because, unlike a trained doctor, they may miss any mass or change in the breast if they perform breast self-examination using the wrong technique or at the incorrect time. Exploring the potential association between breast self-examination and patient delays requires additional research.

Earlier research [7, 31, 32] demonstrated that BC screening behaviours measured by professional breast examination or mammography were related to a lower likelihood of late presentation. At the Breast Disease Treatment Clinic in Sulaimaniyah, regular clinical breast examinations and mammograms are recommended as BC screening measures; therefore, every woman should be aware of this healthcare. However, the reasons why some women do not undergo breast screenings merit additional research.

Fear of receiving a cancer diagnosis or being affected by others was identified as a key finding in the present study. When asked about the exact reason for their fear of a BC diagnosis, these women responded, “BC is fatal with no cure even if it is discovered early,“ “It is a contagious disease,“ and “mastectomy is the only treatment.“ Others claimed that their friends and family had misinformed them or that they had read inaccurate information on BC websites. A multicenter study conducted in Singapore and Malaysia revealed that relatives could have an impact on women’s health decisions and behaviour in both positive and negative ways, depending on whether they incorrectly attribute symptoms to past experiences with benign conditions or promote the use of alternative or conventional medicine as the first line of treatment [33].

Concerning initial symptoms, the present study found that 67.7% of late-presenting women came with a non-painful (non-tender) lump, with a highly significant correlation (p < 0.001). When they felt a lump in their breast, they thought it would go away with time, and they didn’t ask for assistance until the lump got bigger. According to a review, unusual or ambiguous symptoms can increase the probability of delayed presentation in patients with many common malignancies [34]. In a United States study, women with more false beliefs regarding breast lumps were more likely to postpone presentation [35]. Past studies have shown that most patients disregard their symptoms, are unconcerned when they first arise and do not limit their regular activities. This may cause patients to believe it is harmless, delaying their medical care [33, 36].

As predicted, this study found that women with a high degree of awareness about the symptoms of BC were significantly more likely to have an early presentation, indicating that awareness is a significant aspect in determining delay. Furthermore, Richards et al. did a thorough study showing that waiting 3–6 months was strongly linked to more extensive tumours, disease progression, and worse long-term prognoses [37]. In addition, many earlier studies found that the length of time between the first symptom and the pathology-based diagnosis of BC in women with symptoms was a clinically significant risk factor for the later stages of the disease [38, 39]. Our findings were by these studies and revealed that women who presented later than three months had a higher probability of being diagnosed at a later stage than women who presented earlier than three months. For instance, inflammatory BC, bilateral BC, lymph node involvement, GIII, T3, and T4 are more prevalent in late-presenting women.

A unique strength of our study was the careful and complete data collection on patients’ delays and the acquisition of all relevant information from medical records. Instead of asking patients how long the wait was, collecting data on when they first noticed symptoms and when they saw a doctor is more reliable. This study also assessed and analyzed various factors affecting the patient’s behaviour.

This study’s limitations include that most participants were illiterate, married, and housewives; therefore, the results cannot be applied to the entire community. In addition, some patients have passed away due to a terminal illness that arose after their diagnosis and medication. Finally, patients were exposed to BC knowledge during diagnosis and therapy, which may have influenced the results of this study’s assessment of patients’ awareness of the disease.

## Conclusion

We concluded that delayed presentation is a very important health problem in Sulaimani women with BC and is associated with complex interactions between several factors. Almost all these factors demonstrate a deficiency of sufficient knowledge, information, and awareness in our population regarding this fatal disease. Young age, residing in a rural area, low level of education, obesity, being a housewife, smoking, prior benign breast conditions, and women who practice breast self-examinations were all positively linked with late presentation. Furthermore, fear related to the receiving of a cancer diagnosis or the potential impact of others as risk factors for delayed presentation had been established. For instance, doctors can explain that surgery does not cause BC to spread, but that early removal increases the patient’s chance of recovery.

## Data Availability

The datasets used and/or analyzed during the current study are available from the corresponding author upon reasonable request.
